# Changuinola Virus Serogroup, New Genomes within the Genus *Orbivirus* (Family *Reoviridae*) Isolated in the Brazilian Amazon Region

**DOI:** 10.1128/genomeA.00940-13

**Published:** 2013-11-27

**Authors:** Sandro P. Silva, Meik Dilcher, Manfred Weidmann, Valéria L. Carvalho, Alexandre R. Casseb, Eliana V. P. Silva, Keley N. B. Nunes, Jannifer O. Chiang, Lívia C. Martins, Pedro F. C. Vasconcelos, Márcio R. T. Nunes

**Affiliations:** Center for Technological Innovation, Evandro Chagas Institute, Ministry of Health, Ananindeua, Brazila; Department of Virology, University Medical Center, Göttingen, Germanyb; Department of Arbovirology and Hemorrhagic Fevers, Evandro Chagas Institute, Ministry of Health, Ananindeua, Brazilc; Institute of Animal Health and Production, University Rural Federal of Amazon, Belém, Brazild

## Abstract

We report here the first complete genome sequence of a *Changuinola virus* (CGLV) serotype *Irituia virus* (BE AN 28873) isolated from a wild rodent (*Oryzomys goeldi*) in the municipality of Ipixuna, State of Pará, northern Brazil. All genome segments showed similarity with those belonging to members of the genus *Orbivirus*, family *Reoviridae*.

## GENOME ANNOUNCEMENT

The genus *Orbivirus* belongs to the family *Reoviridae* and includes 22 virus species, as well as 10 unclassified or unassigned viruses (1). Orbiviruses are classified based on their morphological, serological, and physicochemical characteristics (2, 3). They are icosahedral, nonenveloped viruses with double-stranded RNA (dsRNA). Their dsRNA genomes consist of 10 segments coding for 7 structural proteins (VP1 through VP7) and at least 3 nonstructural proteins (NS1 through NS3) (4). The serogroup Changuinola has 12 serotypes: *Changuinola virus*, *Almeirim virus*, *Altamira virus*, *Caninde virus*, *Gurupi virus*, *Irituia virus*, *Jamanxi virus*, *Jari virus*, *Monte Dourado virus*, *Ourem virus*, *Purus virus*, and *Saraca virus* (1).

Orbiviruses are transmitted between vertebrate hosts by a variety of arthropod vectors, including midges, ticks, phlebotomine flies, and anopheline and culicine mosquitoes (5). *Bluetongue virus* (BTV) and *African horse sickness virus* (AHSV) are the most economically important members of this genus. Several other orbiviruses are potentially important too, such as *Equine encephalosis virus* (EEV), *Epizootic hemorrhagic disease virus* (EHDV), *Palyam virus* (PALV), and *Peruvian horse sickness virus* (PHSV) (6), while other orbiviruses are causative agents of diseases in humans, such as *Kemerovo virus* (KEMV) and *Tribeč virus* (TRBV) (4).

The Brazilian CGLV serotype *Irituia virus* (BE AN 28873) was isolated at the Evandro Chagas Institute, Belém, Brazil, from the blood of a wild rodent species, *Oryzomys goeldi*, caught on 29 March 1961 in the municipality of Ipixuna, Brazil (3°S 49°W). Antigenic characterization was carried out by complement fixation and neutralization tests (3).

Brain tissue of newborn mice infected with serotype *Irituia virus* was propagated in Vero cells, and after 80% to 90% cytopathic effect (CPE) (3 days postinfection) the supernatant was harvested. Viral particles were concentrated using polyethylene glycol (PEG) centrifugation as previously described (4) and the genomic dsRNA was isolated via a guanidinium isothiocyanate procedure (peqGold Trifast). After DNase treatment to digest cellular DNAs, viral dsRNA was purified from contaminating host single-stranded RNA (ssRNA) by overnight precipitation in 2 M lithium chloride at 4°C as described elsewhere (7).

Furthermore, a self-complementary full-length amplification of cDNAs (FLAC) adapter was ligated to the 3′ ends of each RNA segment (8) to be able to cover the termini. The adapter-ligated dsRNA was converted to cDNA, amplified, and fragmented via the TransPlex whole-transcriptome amplification kit as previously described (4). A pyrosequencing library was prepared (9) and used for sequencing on a GS FLX 454 sequencer (Roche, Life Science) at the Department of Virology, University Medical Center, Göttingen, Germany. In parallel, the same library was sequenced in the Center for Technological Innovation at the Evandro Chagas Institute, Ministry of Health, Brazil.

The assembly of the obtained sequences was done using the GS *de novo* Assembler (Newbler v. 2.6). For further analysis, the software Geneious v. 6.1.4 and a set of programs (SeqMan Pro, SeqBuilder, and Protean) available in the DNASTAR package (Lasergene) were used. The coverage of the assembled genome was 1,522-fold. This is the first report for the complete genome sequence of a Brazilian CGLV serotype.

### Nucleotide sequence accession numbers.

The complete genome sequence has been deposited in GenBank under the accession numbers KF624614 to KF624623.

## References

[1] King AMQ Adams MJ Carstens EB Lefkowitz EJ 2012 Virus taxonomy: classification and nomenclature of viruses, 9th ed. Elsevier, San Diego, CA.

[2] Borden EC Shope RE Murphy FA 1971 Physicochemical and morphological relationships of some arthropod-borne viruses to bluetongue virus a new taxonomic group. Physicochemical and serological studies. J. Gen. Virol. 13:261–271 4333714 10.1099/0022-1317-13-2-261

[3] Karabatsos N 1985 International catalogue of arboviruses. American Society of Tropical Medicine and Hygiene, San Antonio, TX.

[4] Dilcher M Hasib L Lechner M Wieseke N Middendorf M Marz M Koch A Spiegel M Dobler G Hufert FT Weidmann M 2012 Genetic characterization of Tribeč virus and Kemerovo virus, two tick-transmitted human-pathogenic orbiviruses. Virology 423:68–76 22189211 10.1016/j.virol.2011.11.020

[5] Attoui H Mohd Jaafar F Belhouchet M Aldrovandi N Tao S Chen B Liang G Tesh RB de Micco P de Lamballerie X 2005 Yunnan orbivirus, a new orbivirus species isolated from *Culex tritaeniorhynchus* mosquitoes in China. J. Gen. Virol. 86:3409–3417 16298988 10.1099/vir.0.81258-0

[6] Maclachlan NJ Guthrie AJ 2010 Re-emergence of bluetongue, African horse sickness, and other orbivirus diseases. Vet. Res. 41:35 20167199 10.1051/vetres/2010007 PMC2826768

[7] Attoui H Billoir F Cantaloube JF Biagini P de Micco P de Lamballerie X 2000 Strategies for the sequence determination of viral dsRNA genomes. J. Virol. Methods 89:147–158 10996648 10.1016/s0166-0934(00)00212-3

[8] Maan S Rao S Maan NS Anthony SJ Attoui H Samuel AR Mertens PP 2007 Rapid cDNA synthesis and sequencing techniques for the genetic study of bluetongue and other dsRNA viruses. J. Virol. Methods 143:132–139 17433453 10.1016/j.jviromet.2007.02.016

[9] Margulies M Egholm M Altman WE Attiya S Bader JS Bemben LA Berka J Braverman MS Chen Y-J Chen Z Dewell SB Du L Fierro JM Gomes XV Godwin BC He W Helgesen S Ho CH Irzyk GP Jando SC Alenquer MLI Jarvie TP Jirage KB Kim J-B Knight JR Lanza JR Leamon JH Lefkowitz SM Lei M Li J Lohman KL Lu H Makhijani VB McDade KE McKenna MP Myers EW Nickerson E Nobile JR Plant R Puc BP Ronan MT Roth GT Sarkis GJ Simons JF Simpson JW Srinivasan M Tartaro KR Tomasz A Vogt KA Volkmer GA Wang SH Wang Y Weiner MP Yu P Begley RF Rothberg JM 2005 Genome sequencing in microfabricated high-density picolitre reactors. Nature 437:376–380 16056220 10.1038/nature03959 PMC1464427

